# Sleep quality and possible sarcopenia in community-dwelling older adults: physical and mental fatigability as mediators

**DOI:** 10.1186/s12877-025-06751-6

**Published:** 2025-12-26

**Authors:** Tzu-Pei Yeh, Yen-Kuang Lin, Fang-Lin Kuo, Pi-Ju Liu, Li-Chuan Chen, Dorothy Bai, I-Hui Chen

**Affiliations:** 1 School of Nursing, China Medical University, 100 Jingmao Rd., Sec. 1, Beitun Dist, Taichung, 406040 Taiwan; 2 Department of Nursing, China Medical University Hospital, 2 Yude Rd., North Dist, Taichung, 404327 Taiwan; 3 Graduate Institute of Athletics and Coaching Science, National Taiwan Sport University, 250 Wenhua 1 st Rd., Guishan Dist, Taoyuan, 333325 Taiwan; 4 National Center for Geriatrics and Welfare Research, National Health Research Institutes, Miaoli County, 35 Keyan Road, Taiwan Miaoli County 350401 Zhunan Town,; 5 School of Nursing, Center on Aging and the Life Course, Purdue University, West Lafayette, IN USA; 6 Department of Preventive and Community Medicine, Taipei Medical University Hospital, Taipei, Xinyi District 252 Wuxing St, 11031 Taiwan; 7 School of Gerontology and Long-Term Care, College of Nursing, Taipei Medical University, Taipei, Xinyi District 252 Wuxing St, Xinyi District, 11031 Taiwan; 8 School of Nursing, College of Nursing, Taipei Medical University, Taipei, Xinyi District 250 Wuxing St , 11031 Taiwan

**Keywords:** Possible sarcopenia, Sleep quality, Fatigability, Aging, Mediation analysis

## Abstract

**Background:**

Assessment of ‘possible sarcopenia’, introduced by the Asian Working Group for Sarcopenia (AWGS) 2019 guidelines, facilitates earlier identification of at-risk individuals. In this study, we examined the association between sleep quality and possible sarcopenia in community-dwelling older adults, and investigated whether physical and mental fatigability mediate this relationship.

**Methods:**

This cross-sectional study included 200 community-dwelling older adults (mean age 77.52 ± 6.23 years, 48% women) in Taipei, Taiwan. Possible sarcopenia was defined according to AWGS 2019 criteria as low handgrip strength (< 28 kg for men and < 18 kg for women) or poor five-times sit-to-stand test performances (≥ 12 s). Sleep quality was assessed using the Pittsburgh Sleep Quality Index (PSQI; with a score of > 5 indicating poor sleep quality). Physical and mental fatigability were measured using the Pittsburgh Fatigability Scale. After adjusting for age and sex, exploratory mediation analyses examined potential statistical mediation patterns among sleep quality, fatigability dimensions, and possible sarcopenia in these cross-sectional data.

**Results:**

Of the 200 participants, 48% (*n* = 96) met criteria for possible sarcopenia, while 84.5% (*n* = 169) exhibited poor sleep quality. Participants with poor sleep quality showed a significantly higher prevalence of possible sarcopenia than those with good sleep quality (55.6% vs. 6.5%, *p* < 0.001). Normal muscle strength was more prevalent among good sleepers (90.3%) compared to poor sleepers (52.1%) (*p* < 0.001). Participants with possible sarcopenia exhibited significantly higher physical (30.44 ± 7.87 vs. 15.08 ± 5.65, *p* < 0.001) and mental fatigability scores (27.69 ± 8.78 vs. 12.84 ± 6.29, *p* < 0.001) with large effect sizes (Cohen’s d ranged 1.53–2.24). A mediation analysis showed statistical associations between poor sleep quality and possible sarcopenia (B = 0.58, standard error (SE) = 0.14, *p* < 0.001, 95% confidence interval (CI) [0.31, 0.85]). A bootstrap analysis revealed significant indirect effects through physical fatigability (Effect = 0.36, 95% CI [0.20, 0.72]) and mental fatigability (Effect = 0.10, 95% CI [0.01, 0.37]), with physical and mental fatigability accounting for 34.6% and 9.6% of the total statistical effect, respectively.

**Conclusions:**

Poor sleep quality showed strong statistical associations with possible sarcopenia among older adults, with physical and mental fatigability showing significant statistical associations. However, the cross-sectional design limited the ability to infer causal relationships among these variables. While these cross-sectional associations cannot establish causation, screening for sleep quality and fatigability may help identify individuals at risk of sarcopenia before significant functional declines occur, though prospective studies are needed to determine whether interventions targeting both factors could serve as components of sarcopenia prevention strategies.

**Supplementary Information:**

The online version contains supplementary material available at 10.1186/s12877-025-06751-6.

## Introduction

Sarcopenia, characterized by progressive loss of skeletal muscle mass and function, is increasingly recognized as a major health concern in aging populations worldwide [1]. This condition significantly impacts mobility, independence, and quality of life in older adults and is associated with increased risks of falls, fractures, hospitalizations, and mortality [2]. Understanding the biomechanical and physiological factors contributing to sarcopenia is crucial for enhancing functional performance, mitigating injury risk, and optimizing rehabilitation strategies in older adults [3].

The Asian Working Group for Sarcopenia (AWGS) introduced the concept of “possible sarcopenia” in 2019 as a preliminary stage identified by either low muscle strength or poor physical performance, without requiring confirmation of low muscle mass [4]. This new diagnostic category facilitates earlier identification of at-risk individuals in community settings where measuring muscle mass may be impractical or resource-intensive. Early detection of possible sarcopenia is particularly important as it allows for timely interventions before significant functional declines occur. The prevalence of possible sarcopenia in community-dwelling older adults in Asia can be up to 46.0% [5], highlighting its clinical significance as an early detection measure.

Sleep quality is a multidimensional construct encompassing various aspects of sleep, including sleep duration, efficiency, latency, disturbances, use of sleep medications, and daytime dysfunction [6]. Poor sleep quality is particularly prevalent among older adults, with rates exceeding 50% in some populations [7]. Growing evidence suggests associations between poor sleep quality and sarcopenia development through disrupted anabolic hormone secretion [8], systemic inflammation [9, 10], and impaired glucose metabolism [11]. Several studies reported associations of sleep quality with reduced muscle mass, strength, and physical performance in older adults [12, 13], but few have specifically examined the relationship with possible sarcopenia using AWGS criteria.

Poor sleep quality may also indirectly influence muscle function through increased fatigability—defined as the degree of fatigue experienced relative to a standardized activity [14]. Unlike general fatigue, fatigability accounts for activity levels and can be divided into physical and mental dimensions [15]. Studies demonstrate that physical fatigability predicts mobility limitations and mortality [15, 16], while mental fatigability may contribute through decreased motivation and altered neural drive to muscles [17, 18].

Despite these theoretical connections, the mediating role of fatigability in the sleep quality-possible sarcopenia relationship remains unexplored, particularly in Asian populations. This represents a critical knowledge gap, as understanding these pathways could inform targeted interventions to prevent or delay sarcopenia progression. Therefore, in this study, we examined the association between sleep quality and possible sarcopenia in community-dwelling older adults in Taiwan and examined the statistical associations of physical and mental fatigability with this relationship. We hypothesized that: (1) poor sleep quality would be associated with an increased risk of possible sarcopenia; (2) both physical and mental fatigability would be higher in individuals with poor sleep quality; and (3) the relationship between sleep quality and possible sarcopenia would be mediated by physical and mental fatigability.

## Methods

### Study design, settings, and participants

This study, which employed a cross-sectional study design, was conducted from October 2022 to August 2023 in Taipei, Taiwan, using convenience sampling. Convenience sampling was selected for this exploratory study examining mediation pathways, as it facilitated efficient recruitment of participants willing to undergo comprehensive face-to-face assessments, while ensuring adequate statistical power for a mediation analysis. Participants were recruited from community centers. Inclusion criteria were: (1) being aged ≥ 65 years; (2) with a St. Louis University Mental Status Examination score of ≥ 25; (3) with the ability to communicate in Mandarin or Taiwanese; (4) with the capacity to maintain a standing position either independently or with assistive devices; and (5) willing to provide written informed consent. Exclusion criteria were: (1) being unable to ambulate; (2) with a severe behavioral disorder; (3) with cognitive impairment; (4) with significant visual or hearing impairment; and (5) with a speech disorder. An a priori power analysis determined a required sample of 196 participants (f²=0.05, α = 0.05, power = 0.8). An a priori power analysis indicated that 196 participants were required to detect a medium indirect effect (a = 0.26, b = 0.26) using the Sobel test with α = 0.05 and power = 0.80 [19]. Of the 250 individuals initially screened, 20 did not meet the inclusion criteria and 30 declined to participate, resulting in a final sample of 200 participants (response rate: 80%). Given the observed severe imbalance between good (*n* = 31, 15.5%) and poor (*n* = 169, 84.5%) sleep quality groups, a post-hoc power analysis was conducted using SAS Power package (vers. 9.4) to verify the adequacy of the statistical power for between-group comparisons. With the observed effect size (odds ratio = 1.7), α = 0.05, and actual sample size (*N* = 200), the achieved power was 0.99, confirming sufficient statistical power despite the group imbalance.

### Measures

#### Possible sarcopenia

Possible sarcopenia was assessed according to AWGS 2019 diagnostic criteria [4], which define possible sarcopenia solely by abnormalities in muscle strength or physical performance, but without requiring evidence of abnormal muscle mass. In this study, possible sarcopenia was diagnosed when a participant exhibited either low muscle strength or low physical performance. Muscle strength was evaluated using handgrip strength as a proxy. Handgrip strength was measured with a digital dynamometer (Takei TKK 5401, Tokyo, Japan). Participants were assessed in either a standing or seated position, according to their capability and comfort. For the standing protocol, participants stood with feet shoulder-width apart. For the seated protocol, participants sat in a standard-height chair with back support, with their feet flat on the floor; the tested arm’s shoulder was adducted and neutrally rotated, with the elbow flexed at 90° and forearm maintained in a neutral position. In both positions, the dynamometer was held in the dominant hand with the wrist in a neutral position between flexion and extension, as well as between ulnar and radial deviation. Standardized verbal encouragement (“Squeeze as hard as you can. now!“) was provided. Two trials were performed with a 60-s rest interval, and the largest value (in kg) was recorded. According to AWGS (2019) criteria, handgrip strength values below 28 kg for men and below 18 kg for women were classified as low. Physical performance was evaluated using the five-time chair sit-to-stand test (5CSST). Participants were instructed to rise from a seated position in a standard-height chair and return to a seated position five times as quickly as possible without using their arms for support (i.e., with arms crossed over the chest). Timing began when a participant initiated standing and ended after completing the fifth stand, using an HS-70 W-1 timing device (Casio, Japan). A completion time of 12 s or more was classified as low physical performance. Based on these measurements, participants were categorized as having possible sarcopenia (exhibiting abnormal muscle strength or physical performance) or not having possible sarcopenia (exhibiting normal muscle strength and physical performance).

#### Sleep quality

Sleep quality was evaluated using the Pittsburgh Sleep Quality Index (PSQI) [6], a self-reported questionnaire that assesses sleep quality and disturbances over the previous 1-month period. The PSQI comprises seven components (subjective sleep quality, sleep latency, sleep duration, habitual sleep efficiency, sleep disturbances, use of sleep medications, and daytime dysfunction) that sum to a global score ranging 0–21, with higher scores indicating worse sleep quality. A cutoff score of > 5 was used to classify participants as having poor sleep quality [6]. We utilized the PSQI rather than objective measures (polysomnography or actigraphy) for several reasons: (1) resource constraints that made objective sleep monitoring unfeasible for our large community-based sample; (2) the Chinese version of the PSQI (CPSQI) has demonstrated good psychometric properties in Taiwanese samples (sensitivity 98%, specificity 55% at a cutoff of > 5) [20], though we acknowledge the relatively low specificity indicates a substantial false-positive rate that may overestimate poor sleep quality prevalence; and (3) feasibility for community-based research settings. Additionally, while objective measures provide physiological sleep data, the PSQI captures multidimensional sleep experiences including subjective quality and daytime dysfunction that may be relevant to perceived fatigability. In the present study, the scale exhibited good internal consistency (Cronbach’s α = 0.85).

#### Fatigability

Perceived fatigability was assessed using the Pittsburgh Fatigability Scale (PFS), a validated instrument that measures both perceived physical and mental fatigability [14, 21, 22]. The PFS asks participants to indicate their expected level of fatigue after 10 standardized activities. Responses range from 0 (no fatigue) to 5 (extreme fatigue). Separate scores for physical and mental fatigability are computed by summing responses, each ranging 0–50; higher scores indicate greater fatigability. Established thresholds classify scores below 15 for physical fatigability and below 12 for mental fatigability as indicative of low fatigability. In the current study, the PFS demonstrated strong internal consistency, with Cronbach’s α values of 0.87 for the physical and 0.85 for the mental fatigability subscales.

#### Covariates

Potential confounding variables included demographic factors (age, sex, and educational level), body-mass index (BMI), and health status. The health status was further assessed using the number of chronic diseases as diagnosed by a physician and functional independence via the Barthel Index. Depressive symptoms were measured with the 15-item Geriatric Depression Scale [23]. The physical activity level was quantified using the International Physical Activity Questionnaire [24].

#### Data collection

Participants were recruited with collaboration of community centers in Taipei, Taiwan. Community leaders helped identify eligible older adults, and informational sessions were conducted to explain the study purpose and procedures. Eligible participants were invited for a face-to-face assessment that included obtaining informed consent, survey administration, and physical measurements (handgrip strength and 5CSST). Data collection was carried out by trained research personnel in dedicated settings within community centers. Procedures were standardized, and quality control measures (including verification of data completeness) were implemented throughout the assessment sessions.

### Ethical considerations

This study was approved by the Taipei Medical University Institutional Review Board (IRB no. N202204055). Detailed information regarding the study’s purpose, procedures, and potential benefits was provided to all participants, and written informed consent was obtained. Participant confidentiality was maintained through data coding, and personal information was securely stored. All procedures adhered to ethical principles of the *Declaration of Helsinki*, and participants were informed of their right to withdraw at any time.

### Statistical analysis

**Fig. 1:**
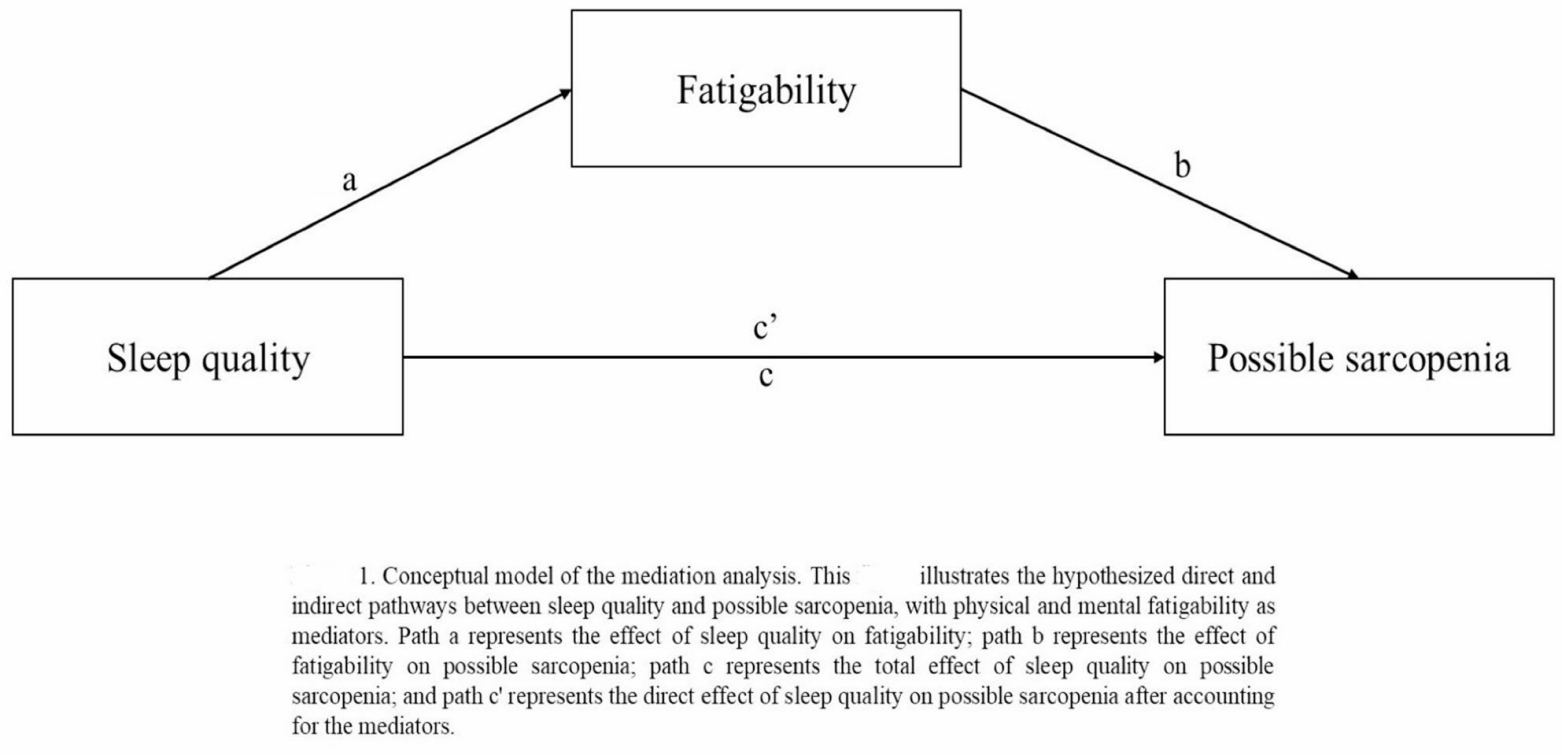
Conceptual model of the mediation analysis. This figure illustrates the hypothesized direct and indirect pathways between sleep quality and possible sarcopenia, with physical and mental fatigability as mediators. Path **a** represents the effect of sleep quality on fatigability; path **b** represents the effect of fatigability on possible sarcopenia; path** c** represents the total effect of sleep quality on possible sarcopenia; and path **c**′ represents the direct effect of sleep quality on possible sarcopenia after accounting for the mediators.

Data analysis was performed using IBM SPSS vers. 28.0 (Armonk, NY, USA) and SAS vers. 9.4 (SAS Institute, Cary, NC, USA). All data underwent double-entry verification to ensure accuracy and consistency. The normality of distributions was confirmed using the Shapiro-Wilk test and visual inspection of histograms. All continuous variables are summarized as the mean and standard deviation (SD), while categorical variables are presented as frequencies and percentages. Between-group comparisons were conducted using independent *t*-tests for continuous variables and Chi-squared/Fisher’s exact tests for categorical variables. We conducted exploratory mediation analysis using the Baron and Kenny approach with bootstrap method to examine statistical associations among sleep quality, fatigability, and possible sarcopenia (Fig. 1). While the Baron and Kenny approach is a classic framework, its application to cross-sectional data can only establish statistical mediation patterns, not causal processes. The mediation model assumes but cannot test temporal ordering in cross-sectional data. Linear regression models were used for continuous mediators (physical and mental fatigability), while a logistic regression was employed for the dichotomous outcome variable (possible sarcopenia). The mediation analysis followed these sequential steps: (1) testing the total effect of the independent variable (sleep quality) on the outcome (possible sarcopenia) using a logistic regression (path c); (2) testing relationships between the independent variable with potential mediators (physical and mental fatigability) using a linear regression (path a); (3) testing relationships between mediators with the outcome while controlling for the independent variable using a logistic regression (path b); and (4) evaluating the direct effect of the independent variable on the outcome while controlling for mediators (path c’). The significance of indirect effects was assessed using bootstrap confidence intervals (CIs) with 5000 resamples. For the mediation analysis, we employed a hierarchical approach to covariate selection. While baseline comparisons identified several potential confounders with significant between-group differences (sex, Barthel Index, physical activity level, and educational level), our final mediation model included only age and sex as covariates. This decision was based on (1) sex showing significant differences between possible sarcopenia groups; (2) age and sex being established biological determinants of sarcopenia in the literature [25], even though age did not significantly differ in our sample; (3) the Barthel Index showing a significant correlation with age (*p* = 0.008), potentially introducing multicollinearity issues; (4) the physical activity level being non-significant when tested in preliminary mediation models and potentially lying on the causal pathway between sleep quality and physical fatigability, thus risking overadjustment bias [26]; and (5) model diagnostics indicating an optimal fit (Hosmer-Lemeshow test: χ²=13.65, df = 8, *p* = 0.091) with this parsimonious approach compared to models with additional covariates. This approach aligns with methodological recommendations to balance confounding control against the risks of overadjustment in mediation analyses [26]. Multicollinearity was assessed using variance inflation factors, with all values being < 5, indicating no significant multicollinearity. To confirm the robustness of our mediation findings, we conducted sensitivity analyses excluding (1) participants with high physical activity levels and (2) participants aged > 82 years (third quartile). Additionally, we assessed sensitivity to unmeasured confounding using the approach by Imai et al. [27], varying the correlation between residuals (ρ) from − 1.0 to + 1.0 to determine the threshold at which indirect effects would become non-significant. Moreover, to examine potential sex differences in the mediation pathways, we conducted sex-stratified mediation analyses. This allowed us to assess whether the mediating roles of physical and mental fatigability differed between women and men. Statistical significance was set to *p* < 0.05, and all tests were two-tailed.

## Results

### Participant characteristics

The study included 200 community-dwelling older adults (mean age 77.52 ± 6.23 years, 48% women) from Taipei, Taiwan. The majority (84.5%, *n* = 169) exhibited poor sleep quality (PSQI > 5), while 15.5% (*n* = 31) had good sleep quality, representing a severe group imbalance. Despite this imbalance, a post-hoc power analysis confirmed adequate statistical power (0.99) for meaningful between-group comparisons. Nearly half of the total sample (48%, *n* = 96) met the AWGS 2019 criteria for possible sarcopenia (for more information see Table 1, left panel).

**Table 1:** Baseline characteristics by sleep quality

Characteristic	Total (*N*=200)	Poor sleep quality (*N*=169)	Good sleep quality (*N*=31)	*p*
Age (years), mean±SD	77.52±6.23	77.55±6.26	77.37±6.13	0.88
Sex, *n* (%)				0.46
Men	104 (52)	86 (50.9)	18 (58.1)	
Women	96 (48)	83 (49.1)	13 (41.9)	
Educational level, *n*(%)				0.03
Uneducated	15 (7.5)	10 (5.9)	5 (16.1)	
Elementary school	66 (33)	56 (33.1)	10 (32.3)	
Junior high school	36 (18)	31 (18.3)	5 (16.1)	
Senior high school	47 (23.5)	45 (26.6)	2 (6.5)	
University or higher	36 (18)	27 (16.0)	9 (29.0)	
Number of chronic diseases, mean±SD	2.4±1.59	2.44±1.63	2.19±1.38	0.38
Barthel Index, mean±SD	97.13±5.41	96.89±5.43	98.39±5.23	0.15
Physical activity level, *n* (%)				0.42
Low	92 (46)	78 (46.2)	14 (45.2)	
Moderate	98 (49)	84 (49.7)	14 (45.2)	
High	10 (5)	7 (4.1)	3 (9.7)	
BMI (kg/m^2^), mean±SD	23.81±3.14	23.96±3.11	22.98±3.20	0.12
Depression, mean±SD	3.79±3.10	3.93±3.01	3.00±3.45	0.17
Sleep quality, mean±SD	8.31±3.38	9.21±2.76	3.39±1.78	<0.001
Physical fatigability, mean±SD	22.45±10.26	23.79±10.36	15.16±5.72	<0.001
Mental fatigability, mean±SD	19.97±10.51	21.28±10.79	12.81±5.69	<0.001
Five-times chair-stand test, *n* (%)				<0.001
Normal	141 (70.5)	110 (65.1)	31 (100)	
Abnormal	59 (29.5)	59 (34.9)	0 (0)	
Muscle strength, *n* (%)				<0.001
Normal	116 (58)	88 (52.1)	28 (90.3)	
Abnormal	84 (42)	81 (47.9)	3 (9.7)	
Possible sarcopenia, *n* (%)				<0.001
Yes	96 (48)	94 (55.6)	2 (6.5)	
No	104 (52)	75 (44.4)	29 (93.5)	

*SD* standard deviation, *BMI* body-mass index

### Baseline characteristics by sleep quality

Baseline characteristics by sleep quality are presented in the middle and right panels of Table 1. Groups were comparable in age, sex, number of chronic diseases, Barthel Index scores, physical activity levels, body-mass index (BMI), and depression scores. However, significant differences were observed in educational levels (*p* = 0.03). Participants with good sleep quality demonstrated significantly better physical performances, with 100% achieving normal results on the 5CSST compared to 65.1% in the poor sleep quality group (*p* < 0.001). Similarly, normal muscle strength was more prevalent among good sleepers (90.3%) compared to poor sleepers (52.1%) (*p* < 0.001). Notably, only 6.5% of participants with good sleep quality presented with possible sarcopenia, compared to 55.6% of those with poor sleep quality (*p* < 0.001).

### Comparison between possible sarcopenia groups

Table 2 presents comparisons of variables between participants with and those without possible sarcopenia. A demographic analysis showed no significant age difference (*p* = 0.36), but revealed a higher proportion of women in the possible sarcopenia group (56.3% vs. 40.4%, *p* = 0.03). Educational levels were comparable between the groups (*p* = 0.07). The groups had similar chronic disease burdens (*p* = 0.63), but those with possible sarcopenia showed significantly lower Barthel Index scores (*p* < 0.001) and lower physical activity levels (*p* = 0.03). BMI and depression scores did not significantly differ.

**Table 2:** Comparison of variables between participants with and those without possible sarcopenia

Variable	Possible sarcopenia (*N* = 96)	No possible sarcopenia (*N* = 104)	*p*	Cohen’s d
Age (years), mean ± SD	77.94 ± 6.23	77.13 ± 6.23	0.36	
Sex, *n* (%)			**0.03**	
Men	42 (43.8)	62 (59.6)		
Women	**54 (56.3)**	**42 (40.4)**		
Educational level, *n* (%)			0.07	
Uneducated	8 (8.3)	7 (6.7)		
Elementary school	32 (33.3)	34 (32.7)		
Junior high school	22 (22.9)	14 (13.5)		
Senior high school	24 (25.0)	23 (22.1)		
University or higher	10 (10.4)	26 (25.0)		
Number of chronic diseases, mean ± SD	2.34 ± 1.58	2.45 ± 1.61	0.63	
Barthel Index, mean ± SD	94.64 ± 6.54	99.42 ± 2.44	< 0.001	
Physical activity level, *n* (%)			0.03	
Low	53 (55.2)	39 (37.5)		
Moderate	38 (39.6)	60 (57.7)		
High	5 (5.2)	5 (4.8)		
BMI (kg/m^2^), mean ± SD	23.74 ± 3.16	23.88 ± 3.13	0.76	
Depression, mean ± SD	3.86 ± 2.80	3.72 ± 3.36	0.74	
Sleep quality, mean ± SD	**10.44 ± 2.71**	**6.34 ± 2.66**	**< 0.001**	**1.53**
Physical fatigability, mean ± SD	**30.44 ± 7.87**	**15.08 ± 5.65**	**< 0.001**	**2.24**
Mental fatigability, mean ± SD	**27.69 ± 8.78**	**12.84 ± 6.29**	**< 0.001**	**1.94**
Five-times chair-stand test, *n* (%)			**< 0.001**	
Normal	38 (39.6)	103 (99.0)		
Abnormal	**58 (60.4)**	**1 (1.0)**		
Muscle strength, *n* (%)			**< 0.001**	
Normal	15 (15.6)	101 (97.1)		
Abnormal	**81 (84.4)**	**3 (3.9)**		

*SD* standard deviation, *BMI* body-mass index

Regarding primary variables, participants with possible sarcopenia had significantly worse sleep quality (10.44 ± 2.71 vs. 6.34 ± 2.66, *p* < 0.001) and higher physical (30.44 ± 7.87 vs. 15.08 ± 5.65, *p* < 0.001) and mental fatigability (27.69 ± 8.78 vs. 12.84 ± 6.29, *p* < 0.001) compared to those without sarcopenia, with large effect sizes (Cohen’s d ranged 1.53–2.24). These unusually large effect sizes for gerontological research are likely inflated by the severe group imbalance in our sample. The 5CSST and muscle strength assessments showed marked differences, with significantly more-abnormal results in the possible sarcopenia group (*p* < 0.001 for both measures). 

### Statistical mediation analysis

As shown in Table 3, the mediation analysis revealed significant direct associations between poor sleep quality and increased physical fatigability (path a_1_: B = 1.39, standard error (SE) = 0.19, *p* < 0.001, 95% CI [1.01, 1.77]) and mental fatigability (path a_2_: B = 1.44, SE = 0.20, *p* < 0.001, 95% CI [1.04, 1.83]). Both models demonstrated moderate explanatory power with respective *R*² values of 0.23 and 0.21. In these analyses, age and sex did not significantly influence either physical or mental fatigability (all *p* > 0.05).

**Table 3:** Mediation analysis of the relationship between sleep quality and possible sarcopenia through physical fatigability and mental fatigability

Path	B	SE	*p*	95% CI
Sleep quality→physical fatigability	1.39	0.19	< 0.001	1.01 ~ 1.77
Sleep quality→mental fatigability	1.44	0.20	< 0.001	1.04 ~ 1.83
Physical fatigability→possible sarcopenia	0.34	0.05	< 0.001	
Mental fatigability→possible sarcopenia	0.26	0.04	< 0.001	
Sleep quality→possible sarcopenia	0.61	0.09	< 0.001	
Direct effect				
Sleep quality→possible sarcopenia	**0.58**	0.14	**< 0.001**	0.31 ~ 0.85
Indirect effect				
Total indirect effect	**0.46**	0.13		0.34 ~ 0.86
Sleep quality→physical fatigability→possible sarcopenia	**0.36**	0.74		**0.20 ~ 0.72**
Sleep quality→mental fatigability→possible sarcopenia	**0.10**	0.61		**0.01 ~ 0.37**

*SE* standard error, *CI* confidence interval. Notes: All models were adjusted for age and sex. Nagelkerk’s *R*² for the full model = 0.84. Confidence intervals that do not contain zero indicate statistically significant indirect effects

Poor sleep quality exhibited a significant direct effect on possible sarcopenia (path c’: B = 0.58, standard error (SE) = 0.14, *p* < 0.001, 95% CI [0.31, 0.85]), indicating that sleep quality was significantly associated with sarcopenia risk independent of mediating factors. When examining specific pathways, both physical fatigability (path b_1_: B = 0.34, SE = 0.05, *p* < 0.001, Exp(B) = 1.41) and mental fatigability (path b_2_: B = 0.26, SE = 0.04, *p* < 0.001, Exp(B) = 1.30) showed significant associations with possible sarcopenia, indicating that for each unit increase in fatigability scores, the odds of possible sarcopenia respectively increased by 41% and 30%.

A bootstrap analysis with 5000 resamples confirmed significant indirect effects, with physical fatigability as a significant statistical mediator in the relationship between sleep quality and possible sarcopenia (Effect = 0.36, SE = 0.74, 95% CI [0.20, 0.72]), and mental fatigability demonstrating a smaller yet statistically significant mediating effect (Effect = 0.10, SE = 0.61, 95% CI [0.01, 0.37]). The total indirect effect through both mediators was 0.46 (SE = 0.13, 95% CI [0.34, 0.86]). When combined with the direct effect (0.58), the total effect of sleep quality on possible sarcopenia was 1.04, with approximately 34.6% of this statistical effect accounted for by physical fatigability and 9.6% accounted for by mental fatigability. The overall model demonstrated excellent fit (Nagelkerke’s *R*²=0.84).

### Sex-stratified mediation analysis

Given the observed sex differences in possible sarcopenia prevalences, we conducted sex-stratified mediation analyses (Supplementary Table S1). While poor sleep quality directly affected possible sarcopenia in both sexes, notable differences emerged in the indirect pathways. Physical fatigability significantly mediated the relationship only in women (indirect effect = 0.36, 95% CI [0.13, 1.05]) but not in men (indirect effect = 0.21, 95% CI [−0.26, 0.87]). Mental fatigability did not significantly mediate the relationship in either sex. These findings suggest that sex-specific mechanisms exist, with physical fatigability playing a more-prominent mediating role in women.

### Sensitivity analyses

Sensitivity analyses confirmed the robustness of our mediation findings (Supplementary Table S2, Supplementary Figure S1). When excluding participants with high physical activity or aged > 82 years, the mediation pathways remained significant with minimal changes in effect estimates. The direct effects remained significant (0.58 and 0.46, respectively), and physical fatigability continued to mediate approximately 34% and 32% of the total effect. An unmeasured confounding analysis showed that indirect effects remained significant until ρ exceeded 0.60, indicating strong robustness to potential unmeasured confounders.

## Discussion

In this cross-sectional study, we investigated the relationship between sleep quality and possible sarcopenia in community-dwelling older adults, with a specific focus on the mediating roles of physical and mental fatigability. While our mediation analyses provide insights into potential statistical pathways linking these variables, the cross-sectional design limits our ability to establish temporal relationships or infer causation. Our findings revealed that poor sleep quality was significantly associated with possible sarcopenia, with physical and mental fatigability showing significant statistical associations in this relationship. While previous studies established associations between fatigue and sarcopenia [28, 29], our study advances this understanding by specifically quantifying the mediating roles of both physical and mental fatigability dimensions in the sleep quality-possible sarcopenia relationship using the AWGS 2019 framework. These findings suggest potential pathways that warrant further investigation and may inform the development of targeted intervention strategies for sarcopenia prevention.

### Prevalences of poor sleep quality and possible sarcopenia

The prevalence (84.5%) we observed substantially exceeded rates reported in previous studies (39%–55%) [30, 31]. While the PSQI cutoff of > 5 had been validated for Taiwanese populations [20], the instrument’s low specificity of 55% means approximately 45% of participants classified as having poor sleep quality may be false positives. This measurement characteristic, combined with urban environmental conditions in Taipei, potential selection bias from community center recruitment, cultural factors influencing sleep behaviors and reporting [32], and the subjective nature of the PSQI, likely contributed to the elevated prevalence.

Our observed prevalence aligns with the upper range reported in Asian populations using AWGS 2019 criteria [5, 33]. Factors contributing to this elevated prevalence include the urban setting’s association with lower physical activity levels and the mean participant age (73.4 years), both of which inherently increase sarcopenia risk. Importantly, the “possible sarcopenia” classification introduced by AWGS 2019 facilitates earlier identification, offering an opportunity for timely interventions prior to significant muscle loss.

### Relationship between sleep quality and possible sarcopenia

The association between poor sleep quality and possible sarcopenia showed remarkably strong effect sizes, which requires careful interpretation. The observed effect size (Cohen’s d = 2.24) substantially exceeds typical findings in gerontology research, where medium to large effect sizes generally range 0.38–0.76 (Hedges’ g) [34]. This unusually large magnitude is likely a consequence of the severe group imbalance (15.5% good vs. 84.5% poor sleep quality), which can inflate effect size estimates in extreme group comparisons [35]. Additionally, the convenience sampling approach that recruited from community centers likely enrolled healthier, more socially active individuals, systematically excluding frail, homebound, or institutionalized older adults who may exhibit different sleep-sarcopenia relationships. This sampling bias may have contributed to both the severe group imbalance and the inflated effect sizes. Therefore, while our findings suggest a notable association between sleep quality and possible sarcopenia, the effect magnitude should be interpreted cautiously and requires replication in more-balanced samples obtained through probability-based sampling methods that include more diverse and representative older adult populations.

Our work advances the field in several ways. Unlike prior studies that focused on individual muscle parameters without established diagnostic thresholds [13], our use of the AWGS 2019 framework advances the field by comprehensively assessing possible sarcopenia. Our findings also complement work by Ye et al. [36], who, by examining sleep quality more comprehensively rather than solely focusing on duration, found that sleeping less than 6 h per night increased the possible sarcopenia risk by over 20% in Chinese adults.

The direct effect of sleep quality on possible sarcopenia remained significant after accounting for fatigability, suggesting multiple pathways through which sleep disruption may influence muscle health. While our cross-sectional data cannot directly assess mechanisms, previous literature suggests potential pathways that may explain the observed associations, including disrupted anabolic hormone secretion [8], systemic inflammation [9, 10], metabolic dysregulation [11], and alterations in body composition favoring fat accumulation over muscle maintenance [37, 38]. These proposed mechanisms remain speculative in the context of our study and require mechanistic research for confirmation. Our findings demonstrate strong statistical associations between poor sleep quality and possible sarcopenia risk. Although our ability to establish causal relationships was limited by the cross-sectional design, alternative causal models are equally plausible, including reverse causation pathways and bidirectional reinforcing cycles among sleep quality, fatigability, and sarcopenia.

### Statistical associations of physical and mental fatigability

A significant contribution of our study is demonstrating statistical mediation effects of physical and mental fatigability in the sleep quality-sarcopenia relationship, extending beyond previous studies that primarily examined direct associations between fatigue and sarcopenia [28, 29]. Although mediation analyses in cross-sectional studies offer valuable insights into potential pathways, they cannot establish temporal order or confirm causality. Physical fatigability, which accounted for approximately 34.6% of the total effect, emerged as a stronger mediator than mental fatigability (9.6%). This difference likely reflects physical fatigability’s more-direct biomechanical and physiological connections to muscle function and activity engagement [15, 39]. Previous research has demonstrated that poor sleep quality is associated with increased injury risk and compromised physical performance in various populations [40, 41], supporting our finding that sleep disruption may contribute to physical functional decline. Sleep disruption is associated with altered skeletal muscle energy metabolism and neuromuscular function, which may relate to increased physical fatigability and reduced physical activity, key factors in muscle maintenance.

Mental fatigability’s significant, albeit smaller, statistical association suggests that cognitive mechanisms also play important roles. Sleep disturbances impair executive function and motivation, potentially increasing perceived effort and reducing physical activity initiation and persistence [42]. Additionally, mental fatigability may influence muscle activation efficiency through central nervous system pathways [43], indicating a more-direct neurophysiological link to muscle performance beyond behavioral effects.

These findings highlight potential synergistic effects between physical and mental fatigability, especially relevant for older adults, where mind-body interactions are critical for physical function. This extends prior research that treated fatigue primarily as a unidimensional construct and suggests the value of multifaceted approaches in future intervention research that target both physical and cognitive fatigability.

### Sex differences in possible sarcopenia

Our finding that women exhibited a higher prevalence of possible sarcopenia (56.3% of women vs. 40.4% of men, *p* = 0.03) aligns with growing evidence of sexual dimorphism in sarcopenia development [25]. However, the sex-stratified findings should be interpreted cautiously as exploratory due to reduced statistical power from smaller subgroup sizes (women *n* = 96, men *n* = 104), which resulted in wider confidence intervals and less stable estimates. Our sex-stratified mediation analyses revealed distinct pathways linking sleep quality to possible sarcopenia between sexes. While poor sleep quality directly contributed to possible sarcopenia risk in both sexes, the statistical association of physical fatigability was significant only in women, accounting for a substantial portion of the total effect. This sex-specific mediation pattern suggests that sleep disruption may impact muscle health through different mechanisms in men and women, though these findings require replication in adequately powered studies. The stronger mediating role of physical fatigability in women may reflect sex-specific physiological responses to sleep disruption. Post-menopausal hormonal changes not only directly affect muscle mass but may also amplify the impact of poor sleep on perceived physical exhaustion [44, 45]. Additionally, women may experience greater sleep-related disruptions in energy metabolism and muscle recovery processes, leading to heightened physical fatigability that subsequently affects muscle function [46]. Although our mediation analyses controlled for sex, these stratified findings highlight the importance of considering sex-specific pathways in sarcopenia prevention strategies. Interventions targeting sleep quality and physical fatigability might be potentially beneficial for women at risk of possible sarcopenia, though these preliminary observations require validation in adequately powered sex-stratified studies before any sex-specific recommendations can be made.

### Physiological mechanisms and geriatric syndromes

The associations observed in this study likely reflect a complex interplay among poor sleep quality, fatigability, and muscle health through multiple biological pathways [15, 39]. Poor sleep quality is associated with both physical and mental fatigability [47, 48], which is associated with reduced physical activity levels [49, 50] and accelerated muscle decline [51], creating a frailty cycle in which these interconnected geriatric syndromes exacerbate each other. Recognizing this dynamic interaction underscores the potential importance of integrated interventions that simultaneously target sleep quality, fatigability, and muscle health to effectively disrupt this cycle and promote healthy aging. This perspective aligns with recent paradigm shifts in preventive health interventions. For instance, Dhahbi et al. [52] argued for moving beyond isolated joint strengthening toward a holistic ‘joint-by-joint’ training approach for injury prevention. Similarly, our findings suggest that sarcopenia prevention may benefit from shifting away from narrow muscle-focused interventions toward integrated strategies that simultaneously address systemic factors including sleep quality, fatigability, and muscle health. Such multifaceted approaches may more effectively disrupt the interconnected cycle of geriatric syndromes and promote comprehensive healthy aging.

### Clinical and public health implications

Our findings have important clinical and public health implications. Routine screening for sleep quality alongside muscle strength and physical performance assessments (e.g., handgrip strength and chair stand tests) is feasible in geriatric primary care and community settings. Early identification of poor sleep could serve as an accessible marker for sarcopenia risk, enabling timely interventions. Community-based programs promoting sleep hygiene education, cognitive behavioral therapy for insomnia (CBT-I), and physical activities tailored to fatigability levels warrant investigation as potential approaches to delay or prevent sarcopenia progression. Specific environmental modifications, such as managing evening blue light exposure, have demonstrated improvements in sleep quality and subsequent physical and cognitive performance [53], suggesting that targeted sleep hygiene interventions addressing modifiable environmental factors could be valuable components of comprehensive sarcopenia prevention programs. Given that physical fatigability accounts for over one-third of the sleep-sarcopenia relationship, such multimodal interventions could reduce the burden of sarcopenia-related disabilities, falls, and healthcare costs. For patients and caregivers, incorporating rest periods, activity pacing, and energy conservation strategies may help mitigate fatigability and maintain functional capacity [54].

### Implications for future research

Our findings provide important foundational evidence for potential pathways linking poor sleep quality with possible sarcopenia. The observed statistical relationships suggest that future sleep intervention studies should consider these associations when designing trials, although the cross-sectional nature of our data requires longitudinal validation to establish true causal mechanisms. The quantified mediation effects (34.6% for physical fatigability and 9.6% for mental fatigability) may inform intervention designs and expected effect sizes for power calculations in future randomized controlled trials, although these estimates should be validated in prospective studies.

Longitudinal studies are needed to establish the temporal sequence and causal relationships of sleep quality and fatigability with possible sarcopenia, including its potential progression to sarcopenia. Such studies should employ stratified sampling techniques to obtain more-representative estimates across diverse older adult populations and investigate potential sex and racial/ethnic differences in these relationships. Randomized controlled trials evaluating sleep hygiene interventions and their impacts on fatigability and possible sarcopenia outcomes would provide crucial evidence for clinical practice, particularly given our finding that physical fatigability appears to be a stronger mediator than mental fatigability. Mechanistic research is essential to elucidate the biological pathways that may underlie the sleep-fatigability-sarcopenia relationship. Studies measuring inflammatory markers (IL-6, TNF-α, and CRP), circadian rhythm disruption indicators, and muscle protein synthesis parameters could clarify how sleep disturbances lead to muscle deterioration through fatigability pathways. Investigation of neuroendocrine factors, including cortisol patterns and growth hormone secretion, would further illuminate the physiological mechanisms connecting sleep quality to muscle health. Clinical translation research should focus on developing and validating fatigability-based screening tools for possible sarcopenia in community settings. This includes determining optimal cutoff values for fatigability measures in possible sarcopenia risk assessments and establishing clinical protocols that integrate sleep quality evaluations into routine geriatric care. Mobile health applications for continuous sleep and fatigability monitoring could support both research and clinical applications in this population

### Strengths and limitations

This study’s strengths include focusing on the emerging concept of possible sarcopenia and simultaneously investigating physical and mental fatigability as mediators with sufficient statistical power. Our findings demonstrated robustness in sensitivity analyses, including exclusion of influential subgroups and assessment of unmeasured confounding. However, several limitations warrant consideration. First, the cross-sectional design precludes causal inferences and temporal ordering verification, which is particularly relevant for mediation analyses. Second, a major limitation is our exclusive reliance on subjective assessments (PSQI for sleep quality and self-reported fatigability) without objective validation. The absence of polysomnography, actigraphy, or performance-based fatigability measures means our findings reflect perceived rather than physiological constructs, introducing potential recall bias, social desirability bias, and interpretation variability. While subjective experiences are clinically relevant as they influence health-seeking behaviors, we cannot determine whether observed associations reflect actual physiological relationships or shared method variance from self-reporting. The PSQI’s specificity of 55% in Taiwanese populations further compounds this limitation, potentially resulting in substantial misclassification. Third, the convenience sampling from community centers likely recruited healthier, more socially active older adults, systematically excluding frail, homebound, or institutionalized individuals. This selection bias limits generalizability to the broader older adult population, particularly more vulnerable subgroups, and contributed to severe group imbalance (84.5% poor vs. 15.5% good sleepers). The observed Cohen’s d values exceeding 2.0 are unusually large for gerontological research and likely represent inflated estimates due to the combined effects of group imbalance and sampling bias rather than true population effects. These findings require replication in more representative samples obtained through probability-based sampling methods. Fourth, although the substantial group imbalance did not compromise statistical power (achieved power = 0.99), it limited our ability to interpret findings from the smaller group with good sleep quality and may have further inflated effect size estimates. Finally, although we adjusted for several confounders and conducted sensitivity analyses, residual confounding from unmeasured variables (e.g., nutritional status, specific medications) cannot be ruled out. However, our sensitivity analyses indicated that unmeasured confounding would need to explain more than 70% of residual variance to nullify the observed mediation effects, suggesting our findings are robust to potential unmeasured confounders. The reduced sample sizes in the sex-stratified analyses also resulted in wider confidence intervals, limiting the precision of sex-specific effects estimates.

## Conclusions

This study provides novel evidence that poor sleep quality shows strong statistical associations with possible sarcopenia in community-dwelling older adults, with physical and mental fatigability showing significant statistical associations in this relationship. While these cross-sectional associations provide hypothesis-generating evidence, longitudinal validation is essential before any causal inferences can be drawn. Our findings suggest potential associations that may inform future research on interventions targeting both sleep and fatigability as possible components of sarcopenia prevention strategies. The sex-specific patterns observed should be considered exploratory given the limited subgroup sizes.

## Supplementary Information

Supplementary Material 1.

## Data Availability

The data that support the findings of this study are not publicly available due to privacy and ethical considerations but are available from the corresponding author upon reasonable request.
