# Supplementary material for: Sleep quality and possible sarcopenia in community-dwelling older adults: physical and mental fatigability as mediators
Source: BMC Geriatr. 2025 Dec 26;26:230. doi: 10.1186/s12877-025-06751-6 (PMC12911209; doi:10.1186/s12877-025-06751-6)
Supplement: Supplementary file 1 — Supplementary Material 1. [file 12877_2025_6751_MOESM1_ESM.docx]

| Supplementary Table S1. Sex-stratified mediation analysis of the sleep quality-sarcopenia relationship through fatigability | | | | | |
| --- | --- | --- | --- | --- | --- |
| **Sex** | **Path** | **B** | **SE** | ***p*** | **95% CI** |
| Women |  |  |  |  |  |
|  | Sleep quality→physical fatigability | 1.19 | 0.26 | <0.001 | 0.66~1.71 |
|  | Sleep quality→mental fatigability | 1.16 | 0.29 | <0.001 | 0.59~1.73 |
|  | Physical fatigability→possible sarcopenia | 0.30 | 0.09 | 0.001 |  |
|  | Mental fatigability→possible sarcopenia | 0.04 | 0.06 | 0.505 |  |
|  | Sleep quality→possible sarcopenia | 0.77 | 0.16 | <0.001 |  |
|  | Direct effect |  |  |  |  |
|  | Sleep quality→possible sarcopenia | 0.72 | 0.22 | 0.001 | 0.28~1.16 |
|  | Indirect effect |  |  |  |  |
|  | Total indirect effect | 0.46 | 1.34 |  | 0.34~0.86 |
|  | Sleep quality→physical fatigability→possible sarcopenia | 0.36 | 7.47 |  | 0.13~1.05 |
|  | Sleep quality→mental fatigability→possible sarcopenia | 0.05 | 1.02 |  | -0.07~0.33 |
| Men |  |  |  |  |  |
|  | Sleep quality→physical fatigability | 1.51 | 0.27 | <0.001 | 0.97~2.06 |
|  | Sleep quality→mental fatigability | 1.63 | 0.27 | <0.001 | 1.10~2.16 |
|  | Physical fatigability→possible sarcopenia | 0.35 | 0.07 | <0.001 |  |
|  | Mental fatigability→possible sarcopenia | 0.38 | 0.08 | <0.001 |  |
|  | Sleep quality→possible sarcopenia | 0.43 | 0.18 | <0.001 |  |
|  | Direct effect |  |  |  |  |
|  | Sleep quality→possible sarcopenia | 0.39 | 0.17 | 0.019 | 0.07~0.72 |
|  | Indirect effect |  |  |  |  |
|  | Total indirect effect | 0.48 | 4.49 |  | 0.30~1.28 |
|  | Sleep quality→physical fatigability→possible sarcopenia | 0.21 | 3.09 |  | -0.26~0.87 |
|  | Sleep quality→mental fatigability→possible sarcopenia | 0.27 | 2.85 |  | -0.07~1.15 |

Notes: SE, standard error; CI, confidence interval. Indirect effects are considered significant when the 95% CI does not include zero.

Notes: SE, standard error; LLCI, lower limit confidence interval; ULCI, upper limit confidence interval.

| Supplementary Table S2. Sensitivity analysis for mediation model robustness | | | | | |
| --- | --- | --- | --- | --- | --- |
| **Analysis** | **Effect type** | **Effect** | **SE** | **LLCI** | **ULCI** |
| Excluding high physical activity |  |  |  |  |  |
|  | Direct effect | 0.58 | 0.14 | 0.30 | 0.85 |
|  | Total indirect effect | 0.42 | 4.43 | 0.30 | 0.80 |
|  | Through physical fatigability | 0.34 | 2.67 | 0.16 | 0.68 |
|  | Through mental fatigability | 0.08 | 1.77 | 0.01 | 0.33 |
| Excluding age >82 years (Q3) |  |  |  |  |  |
|  | Direct effect | 0.46 | 0.14 | 0.19 | 0.77 |
|  | Total indirect effect | 0.42 | 5.29 | 0.29 | 0.80 |
|  | Through physical fatigability | 0.32 | 5.29 | 0.10 | 0.66 |
|  | Through mental fatigability | 0.10 | 0.16 | 0.00 | 0.41 |

| 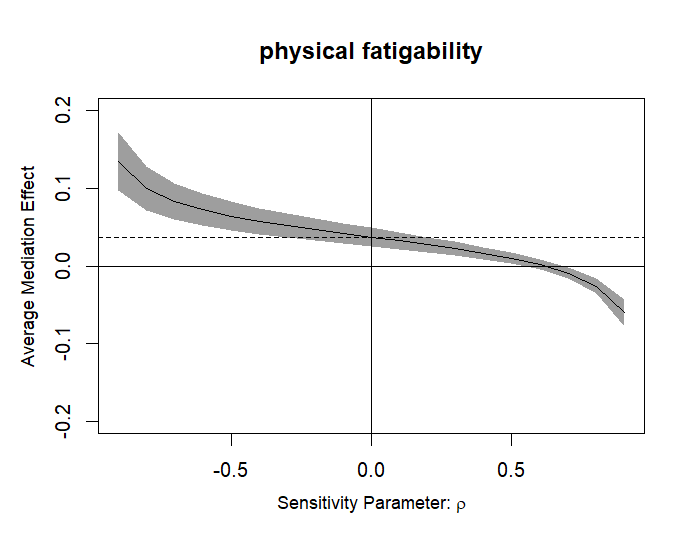 |
| --- |
| **Panel A. Physical fatigability mediation model.** A sensitivity analysis was used to examine the robustness of the indirect effect of sleep quality on sarcopenia through physical fatigability to unmeasured mediator-outcome confounding. The mediation effect remained statistically significant (confidence interval excluding zero) until ρ>0.70, indicating that unmeasured confounding would need to account for more than 70% of the residual variance in both mediator and outcome models to nullify the observed mediation effect. |
| 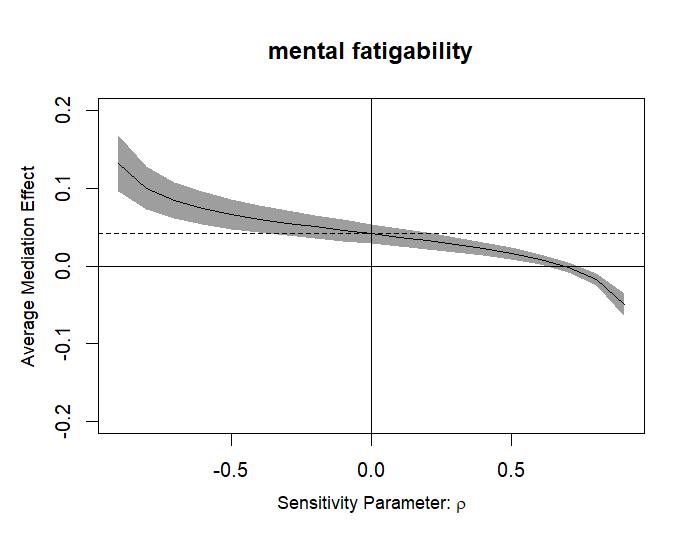 |
| **Panel B. Mental fatigability mediation model.** A sensitivity analysis was used to examine the robustness of the indirect effect of sleep quality on sarcopenia through mental fatigability to unmeasured mediator-outcome confounding. The mediation effect remained statistically significant (confidence interval excluding zero) until ρ>0.60, indicating that unmeasured confounding would need to account for more than 60% of the residual variance in both mediator and outcome models to nullify the observed mediation effect. |
| Supplementary Figure S1. Sensitivity analysis for unmeasured confounding in mediation models. |
